# Pruritic folliculitis of pregnancy*

**DOI:** 10.1590/abd1806-4841.20164735

**Published:** 2016

**Authors:** Lilian Mathias Delorenze, Letícia Guedes Branco, Luiza Fiszon Cerqueira, Wellington Batista Vasques, Simone de Abreu Neves Salles, Enoi Guedes Vilar

**Affiliations:** 1 Universidade Federal Fluminense (UFF) – Niterói (RJ), Brazil; 2 Universidade do Estado do Rio de Janeiro (UERJ) – Rio de Janeiro (RJ), Brazil; 3 Universidade Federal do Rio de Janeiro (UFRJ) – Rio de Janeiro (RJ), Brazil

**Keywords:** Folliculitis, Pregnancy, Prurigo

## Abstract

Pruritic folliculitis of pregnancy is a rare disease of unknown etiology. It occcurs primarily during pregnancy, usually with spontaneous resolution postpartum. It is characterized by a benign dermatosis, with papular and pustular follicular lesions that first appear on the torso and occasionally spread throughout the body. We report the case of a patient in the 27th week of pregnancy, with a two-month evolution of pruritic and papular erythematous lesions on her lower back. Differential diagnosis includes other pregnancy-specific dermatoses: gestational pemphigoid, pruritic urticarial papules and plaques of pregnancy (PUPPP), prurigo of pregnancy, and (PUPPP) and prurigo of pregancy. Histopathological tests showed changes consistent with pruritic folliculitis of pregnancy. This case is relevant due to its rare nature and its clinical and histopathological characteristics.

## INTRODUCTION

Pruritic folliculitis of pregnancy (PFP) is a pregnancy-specific dermatosis with follicular lesions (papules and pustules) on the torso or spread throughout the body.^1-7^ Described by Zoberman and Farmer (1981), this dermatosis develops in the second or third trimester of pregnancy, i.e., from the fourth to the ninth month.^2,3,6^ It affects both gilts and multiparous women. The symptoms last between two and three weeks, with spontaneous regression after delivery. Its exact etiology is unknown, especially due to its rare occurrence. No immunohistological patterns have been discovered, and its histopathology is non-specific inflammatory folliculitis. Apparently, this disease causes no other maternal or fetal complications.

## CASE REPORT

**Figure 1:**
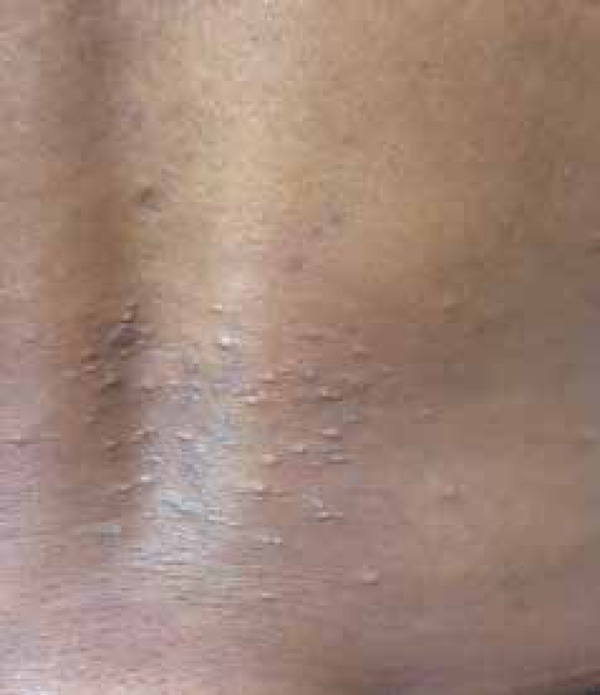
Lesions on the lower back

**Figure 2:**
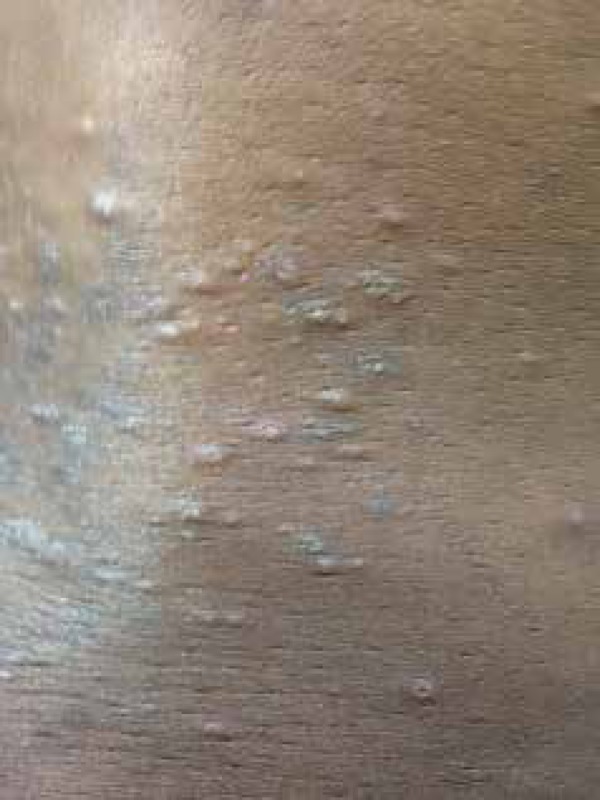
Lesions on the lower back

**Figure 3:**
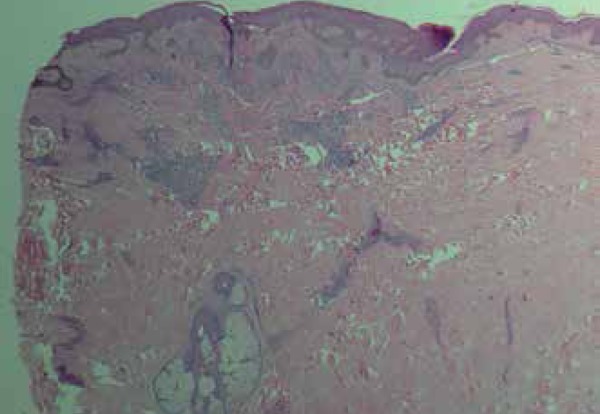
Dermis with inflammatory infiltrate consisting of small and medium-sized mononuclear cells, located around the vessels and in the topography of hair follicles

**Figure 4:**
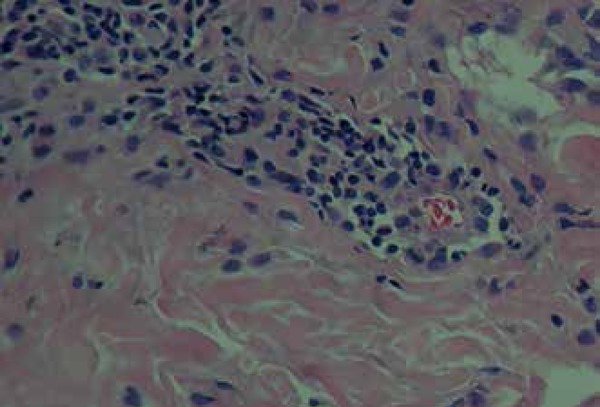
A discrete leukocytoclasia and cells with slightly beveled cores

A 39-year-old black multiparous female, 27 weeks pregnant, reported a 2-month evolution of pruritic papules on her lower back. She denied similar episodes in her previous pregnancies (G3 P2 A0) and previous treatments. Physical examination revealed grouped erythematous papules, some of which were pustular, and others showed central crusts in the lumbar region (Figures 1 and 2). An incisional biopsy was performed on one of the lesions on the lower back. Histopathology showed epidermis with mild psoriasiform acanthosis, dermis with wedge-shaped inflammatory infiltrate consisting of small and medium-sized mononuclear cells, located around the vessels and in the topography of hair follicles (Figure 3). Discrete leukocytoclasia and cells with slightly beveled cores were also found (Figure 4). Immunohistochemistry was positive for myeloperoxidase, HAM56, lysozyme, and CD3. The changes found were consistent with pruritic folliculitis of pregnancy. Benzoyl peroxide was prescribed, but the patient opted to not take medication during pregnancy. The lesions regressed spontaneously about one to two months after delivery.

## DISCUSSION

Pruritic folliculitis of pregnancy is a benign pruritic dermatosis of pregnancy characterized by follicular papules and pustules on the torso or spread throughout the body. It typically develops in the second quarter of pregnancy^1-7^, and the incidence rate is approximately 1 in 3,000 pregnancies.^7^ Its etiology is uncertain, probably due to its rare ocurrence.^1,6,7^ A few studies on the subject have been conducted, and a few cases have been reported in the literature. It is believed that many cases are under-reported, as it is often confused with bacterial folliculitis.^1,7^

The lesions usually consist of small (3-5mm) erythematous papules on the upper torso, which might spread in form of follicular erythematous papules and pustular lesions. Prurigo often occurs and can occasionally be extremely severe.^6.8^

Its histopathology is non-specific, showing characteristics of acute folliculitis, as well as intraluminal pustules with neutrophils, lymphocytes, macrophages, and occasional eosinophils. The dermis shows superficial edema with perivascular lymphocytic infiltrate and some eosinophils. Staining for microorganisms was negative, revealing sterile folliculitis.^8^

Differential diagnoses include other pregnancy-specific dermatoses (e.g. PUPPP, prurigo of pregnancy, and gestational pemphigoid).^1-7^ The absence of bullous lesions accompanied by non-specific histopathology, as well as negative immunofluorescence, might help differentiate pruritic folliculitis of pregnancy from gestational pemphigoid. PUPPP may be distinguished from PFP by the absence of pregnancy stretch marks and skin rashes, typical of PUPPP. Unlike the PFP, the prurigo of pregnancy shows non-follicular lesions and usually develops on the extremities.^7^

On the other hand, the differential diagnosis should include papulopustular eruptions that coincide with pregnancy (e.g., acne, bacterial folliculitis). Some characteristics that distinguish PFP from acne are the absence of comedones, the presence of prurigo and no lesions on the face.^7^

Treatment consists of topical medication such as low-potency corticosteroids, 10% benzoyl peroxide or a mix of both, or UVB therapy.^1,2,3,7^ Other drugs have been tested, such as topical antifungal medications and topical or systemic antibiotics, but they have shown minimal effects. If the lesions are asymptomatic, their natural resolution after delivery should be considered.^1,7,8^

The lesions of pruritic folliculitis of pregnancy develop during pregnancy and disappear within weeks after delivery.^2^ The maternal prognosis is good, and the recurrence in subsequent pregnancies is exceptional. Fetal prognosis is good, usually without adverse effects to the newborn.^1,7^

This case showed clinical, histopathological, and evolutionary manifestations consistent with PFP. As described in the literature, the lesions regressed completely after delivery, requiring no treatment, which should be considered depending primarily on the presence of symptoms such as prurigo. In the case reported here, the patient opted out of treatment because the disease showed mild symptoms, and she was concerned about the use of medications during pregnancy. The complete involution of the disease occurred postpartum, as described in the literature.
